# Decoding the tuberculosis puzzle: mechanical factors driving disease progression

**DOI:** 10.3389/ftubr.2025.1570292

**Published:** 2025-03-28

**Authors:** Pere-Joan Cardona

**Affiliations:** ^1^Microbiology Department, Northern Metropolitan Clinical Laboratory, Hospital Universitari “Germans Trias i Pujol”, Badalona, Catalonia, Spain; ^2^Experimental Tuberculosis Unit, Germans Trias i Pujol Research Institute (IGTP), Badalona, Catalonia, Spain; ^3^Centro de Investigación Biomédica en Red de Enfermedades Respiratorias (CIBERES), Madrid, Spain; ^4^Genetics and Microbiology Department, Autonomous University of Barcelona, Cerdanyola del Vallès, Spain

**Keywords:** tuberculosis pathogenesis, *Mycobacterium tuberculosis* evolution, pulmonary TB tropism, pediatric TB paradox, immune response modulation

## Abstract

It is stated that, following infection with *Mycobacterium tuberculosis* (Mtb), only 5–10% of individuals will develop active tuberculosis (TB), predominantly in the pulmonary form. After excluding major comorbidities that impair immune responses—such as undernourishment, alcohol abuse, smoking, HIV infection, and diabetes—there remains no clear explanation for this progression. Extensive efforts have been made to identify a transcriptomic biosignature in blood to predict disease development, yet none have been successful. This perspective aims to provide insights into this phenomenon. In adults, pulmonary TB exhibits a particular tropism for the upper lobes, primarily due to localized mechanical factors. Reduced mobility exacerbates the neutrophilic inflammatory response fuelling Mtb extracellular growth, while gravitational stress impairs the function of secondary lobular septa, hampering lesion encapsulation. Interestingly, such tropism is absent in children, as these regional differences do not exist. Instead, they develop self-healing, small lesions known as Ghon foci. However, children have a significantly higher likelihood of developing disseminated extrapulmonary TB, a phenomenon that could be named as the pediatric TB paradox. This has traditionally been attributed to an immature immune response, but an alternative explanation may lie in the profound modifications occurring in lung parenchyma and microvascular maturation during the first 2 to 3 years of life. Ultimately, the evolution of Mtb suggests an original symbiotic relationship with humans, which has been disrupted by socio-demographic and cultural factors. These shifts may have transformed Mtb from a natural enhancer of Th1 responses and trained immunity into the leading infectious killer of humankind.

## 1 Primary or atypical TB?

One of the major challenges of *Mycobacterium tuberculosis* (Mtb) infection is to predict its capacity to progress toward pulmonary tuberculosis disease (TB). After the Second World War and the active TB surveillance, Stead developed the “unitary concept” (1). This concept arose from the observation that most children exhibited calcifications of old TB lesions in multiple locations within the lung parenchyma and the hilar lymph nodes, which was termed “primary TB.” In contrast, TB lesions in adults were primarily located in the upper lobes and were referred to as “post-primary TB.” The “Unitary concept” postulated that “once infected, always infected” and that “reinfection was never possible.” It suggested that the initial infection could disseminate until an immune response occurred, and TB development in adults resulted from the reactivation of a childhood lesion due to immunodepression. However, it is now well established that reinfection can occur at any time and may cause TB disease in adults, particularly in settings with a high risk of infection (2). This mechanism was already described long time ago, with TB development being attributed to successive reinfections of the upper pulmonary lobes, as explained by Pottenger and Canetti (3, 4).

Furthermore, this knowledge led to the classification of typical and atypical TB radiographic patterns in adults. Radiographs are considered typical if they show either an upper lobe infiltrate or a cavitary lesion in the upper lung zones. The presence of lymphadenopathy, a lower or middle lobe infiltrate, or pleural effusion in conjunction does not alter the classification as typical. Conversely, atypical radiographs are characterized by lymphadenopathy, lower or middle lobe infiltrates, or effusions without the presence of either a cavity or upper lobe infiltrate. HIV positivity is strongly associated with atypical radiography, thus being linked to the integrity of the immune response (Supplementary Figure 1). However, the interval between infection acquisition and TB development does not predict the radiographic pattern in adults, thereby challenging the primary/post-primary classification. Despite this, the terms are still used synonymously with atypical/typical radiographic presentations (5).

In the last 20 years, significant efforts have been made to discern why Mtb infection leads to TB. These efforts have generated an extraordinary body of knowledge aimed at identifying immunological markers that predict this progression. Notably, the work of the O'Garra group has marked a significant milestone in this field, providing a strong signature capable of distinguishing between Mtb infection and TB (6). Since then, substantial investment has been made in TB prognosis through transcriptomics, though this has not yielded a highly predictive value. In fact, a recent study by Mendelsohn et al. (7), analyzing the ability of various transcriptomic signatures to predict TB progression in close contacts of TB cases, concluded that these signatures did not outperform the IGRA assay (Quantiferon Gold). A fundamental issue in this research is that TB development is a local pulmonary process, whereas biomarker discovery has primarily relied on blood specimens. This has well-documented implications, such as the well-known anergy phenomenon (8).

## 2 The pediatric paradox

Overall, these studies have provided important insights into TB that are not widely considered in infants, primarily due to the challenges of clinical sampling, which complicate pediatric TB management. Monitoring Mtb-infected populations (9, 10) has provided strong evidence of the exceptionally high risk of developing TB in children under 5 years of age who are infected with Mtb (almost 40%) and even in those aged 5 to 14 years (20%). This fact is widely recognized in TB guidelines, which emphasize, for instance, the need for chemoprophylaxis (11) or BCG vaccination in neonates in high-risk settings (12).

Interestingly, when trying to understand the mechanism underlying this phenomenon, the usual explanation appeals to the immunological immaturity of children. However, for example, in perinatal TB, tuberculin or IGRA test become positive, although too late to be useful for diagnosis purposes (13). Furthermore, in countries where pediatric immunization schedules are feasible, vaccinations typically commence at 2 months of age, often with nine different vaccines administered simultaneously (14). This suggests that, by this age, the immune system is already capable of mounting a robust defense against parasitic microorganisms.

### 2.1 Explaining the tropism of TB development in the upper lobes

Several hypotheses have been proposed regarding the tropism of adult TB for the upper lobes. Initially, it was thought that the reduced mobility of this pulmonary region and the high oxygen pressure might favor Mtb growth (15, 16). However, this perspective has evolved. Current understanding suggests that the lack of mobility in the upper lobes leads to decreased capillary perfusion and, consequently, the surveillance capacity of the lymphatic system, allowing local accumulation of parasites (17). This accumulation promotes the induction of a neutrophil-based inflammatory response, which in turn facilitates rapid extracellular Mtb growth (18) and a sudden expansion of the lesion (19), in a process resembling the formation of soap bubbles (20).

This rapid enlargement of the infection site and the sustained inflammatory response are crucial in overcoming another protective mechanism. This is the encapsulation by fibroblasts present in the interlobular septa. These septa transmit the diaphragm's contraction to the entire parenchyma and isolate groups of acini within secondary pulmonary lobules, which are ~2 cm in diameter (21). In a TB experimental model using mini-pigs, which possess a human-like pulmonary structure, encapsulation of Mtb lesions is almost complete within 1 week (22). This suggests that the inflammatory process is highly acute; otherwise, lesion expansion would be inhibited.

Despite the ongoing controversy regarding the proinflammatory capacity of ancient (L1, 5, and 6) versus modern lineages (L2–4)—with some studies suggesting higher proinflammatory potential in ancient lineages (23) and others indicating the opposite (24)—it ultimately appears that TbD1 deletion confers significantly greater virulence to Mtb. This increased virulence is reflected in a higher bacillary load (25) and larger lesions (26) in modern lineages.

Similarly, although transmission rates were comparable, an ancient lineage was less likely to progress to active tuberculosis than a modern one in a cohort of HIV-negative, BCG-vaccinated contacts with a median age of 16 years (interquartile range: 8–25 years) (27).

Taken together, these findings suggest that modern lineages are less effectively recognized and eliminated by the host immunity, allowing for enhanced local dissemination and the development of more extensive lesions, thereby compromising the effectiveness of mechanical barriers (28).

Interestingly, the size of the secondary pulmonary lobules is not homogeneous. They are larger in the upper lobes because the alveoli in this region have a substantial amount of lung tissue beneath them, allowing gravity to exert a greater force pulling the lung away from the pleural space compared to basal alveoli. Consequently, at a normal lung volume, alveoli at the apex reach full capacity during inspiration, directing most of the inspired air toward the base, where alveoli are more compliant and less extended (29). Furthermore, the constant stretch of the septa in the apex reduces the fibroblasts' reactivity to mechanical changes (30).

Overall, multiple factors convergence in the upper lobes to promote rapid Mtb infection progression. Firstly, reduced parasite surveillance facilitates bacterial accumulation and neutrophile infiltration, promoting rapid extracellular Mtb growth. Secondly, the large alveolar volume in this region provides ample space for lesion expansion without stimulating fibroblasts in the septa, thereby limiting encapsulation. Thirdly, fibroblasts in the apex remain in a constant state of stretch, reducing their responsiveness to mechanical changes and further delaying lesion encapsulating.

**Figure 1:**
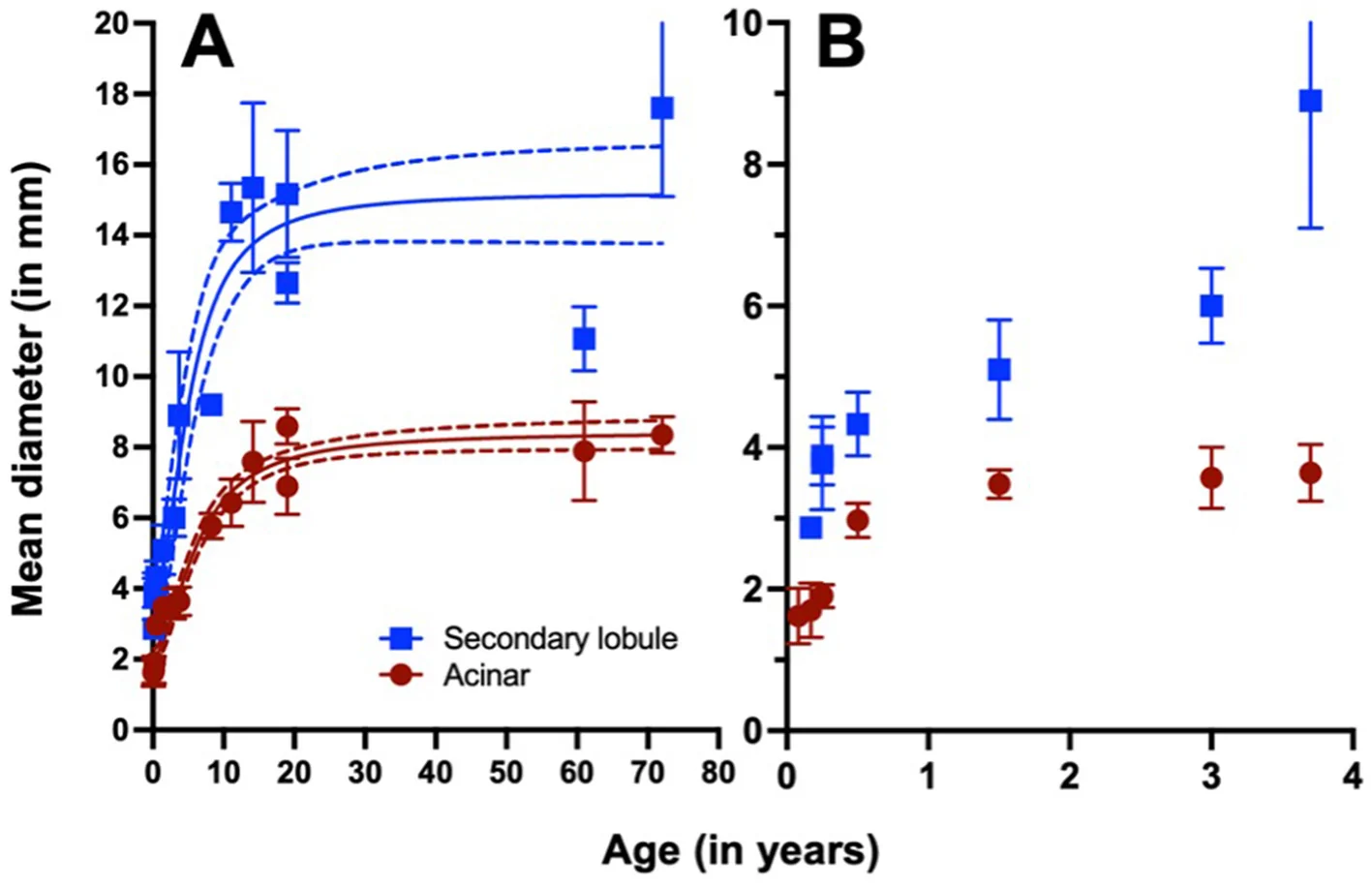
Relationship between acinar and secondary lobule diameter and age. Data show the mean and standard deviation. The curves represent a sigmoidal fit, while the dotted curves indicate the 95% confidence interval, with an *R*^2^ of 0.914 for acinar and 0.855 for secondary lobule measurements (GraphPad Prism, vs 10.1.0). Data are presented globally **(A)** and in detailed up to the age of four **(B)**. Raw data were obtained from Osborne et al. (31).

Interestingly, the size of secondary lobules changes with age. Data obtained from Osborne et al. (31) (Figure 1) illustrate how the size of secondary lobules increases drastically up to the age of ten, at which point it stabilizes.

According to these data, the risk of TB progression in infancy should be lower, as the acini are less inflated, and are closer to the interlobular septa, increasing the likelihood of encapsulating and curtailing lesion growth.

### 2.2 Pediatric TB: is it different disease?

The natural history of childhood intrathoracic TB has been well described through radiological monitoring in the pre-chemotherapy era and reviewed by Marais et al. (32) (Supplementary Figure 2). This monitoring highlights the risk of dissemination, leading to meningitis or miliary TB in infants under 1 year of age, as well as the predominance of lymph node enlargement up to the age of ten.

**Figure 2:**
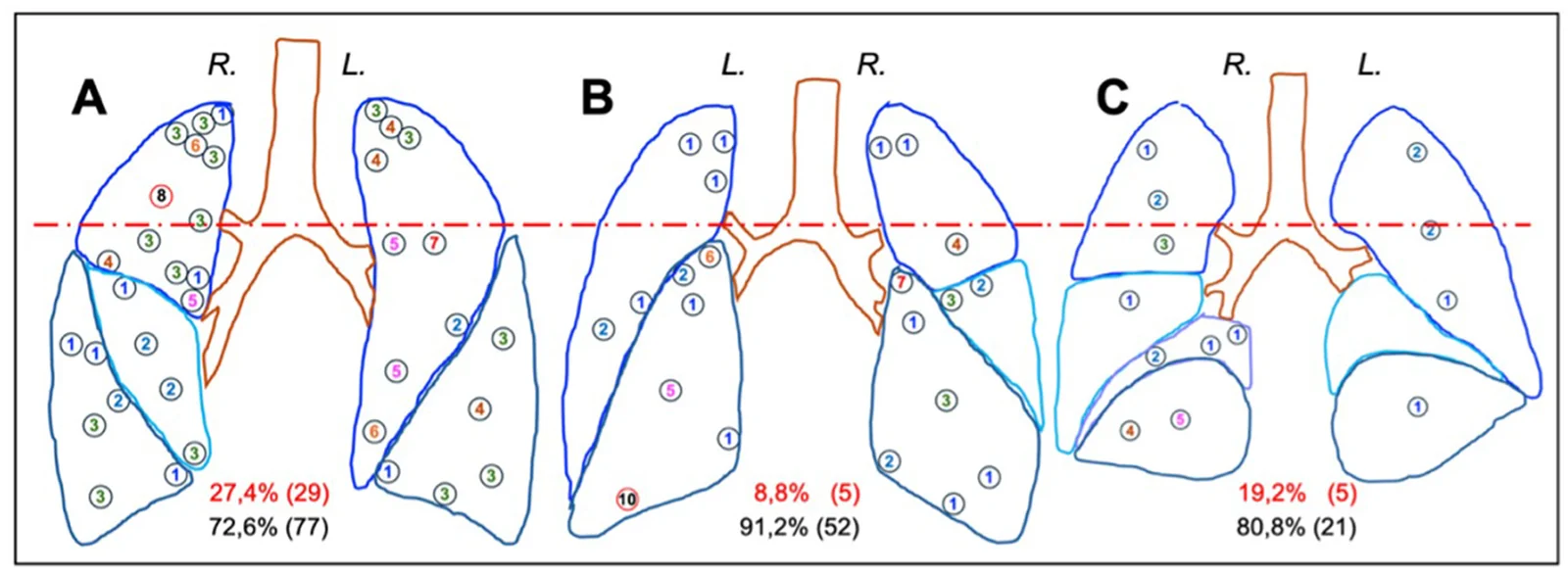
Distribution of primary foci described in the Anton Ghon's studies of primary TB in children. The images correspond to anterior **(A)**, posterior **(B)** and medial **(C)** pulmonary projections. Circles indicate the position and number of the foci. The right and left lungs are marked as *R* and *L*, respectively. The dotted red line categorizes the upper and medium-lower regions of the pulmonary lobes, within the percentage of foci is provided in parentheses, with red indicating the upper region and black indicating the middle-lower region. Data were obtained from Donald et al. (33).

Up to the age of two, one of the pathological hallmarks are the parenchymatous infiltration, with the Ghon focus typically accompanied by lymphadenopathy, forming the so-called “Ghon complex.” The size of the Ghon focus was described using a somewhat rudimentary “agricultural” classification, including comparisons to a pea (10 mm), hazelnut (10–15 mm), and cherry (20 mm) as the maximum. According to Osborne et al., this corresponds to the occupation of ~two to three secondary lobules (31). This lesion is typically self-limiting (Figure 2) unless bronchial dissemination occurs. In most cases, it resolves with calcification, forming the Ranke complex, with is of a smaller size.

Figure 2 illustrates the distribution of the Ghon focus on primary TB in children. Nodules are distributed homogeneously, with 20.6% (39 foci) located in the upper regions, corresponding to the relative volume of this zone, and 79.4% (150 foci) in the remainder of the lungs. The anterior upper portions of the upper lobes are affected in particular, though the highest concentration of foci is observed in the posterior lower portions of the lower lobes. Notably, the right lung contains more foci than the left, as described by Ghon (33). Interestingly, even when lesions are located in the upper regions, their progression is similar to those in the middle or lower regions. These data suggest a protective role of the secondary lobule septa, which, due to the reduced diameter of the acini and its close vicinity (Figure 1), may facilitate encapsulation of the foci in infants.

During the pre-chemotherapy era, Murray described Ghon focus as an initial lesion eliciting a non-specific inflammatory response, predominantly characterized by a neutrophilic infiltration, which was later replaced by mononuclear and lymphocytic cells. Caseation occurred within a few weeks, with similar changes observed in the draining lymph nodes. Six weeks post-infection, the tuberculin test became positive, and encapsulation followed, leading to calcification within 5 to 15 months, even if radiographic evidence after 1 year. Murray found that the prognosis of primary TB was generally excellent, with fatal outcomes occurring only in a small proportion of young children shortly after infection. He attributed these severe cases to children exposed to heavy and continuous infectious aerosol inhalation. Such outcomes were also observed in adolescents and adults (34).

It is important to note that primary TB lesions are not exclusive from infancy, as highlighted by Woodring et al. (35). Supplementary Figure 3 illustrates the impact of primary lesions in adults, where they are less common but typically characterized by patchy upper lobe lesions that cavitate in ~50% of cases. These lesions are often accompanied by bronchial dissemination, presenting as a “tree-in-bud” pattern (36, 37), and a continuous generation of daughter micronodules surrounding the primary lesion. This process has been crucial in the generation of active TB lesions (38).

### 2.3 Can pulmonary development explain the high haematogenous risk of dissemination in pediatric TB?

Overall, it appears that the protective scenario in infants, due to the smaller size of the acinus and its close relationship with the interlobular septa, results in limited lesions that tend to resolve through calcification. However, there remains a significant risk of haematogenous dissemination, potentially leading to a miliary TB and/or meningitis, both of which are highly fatal forms of the disease. In this context, greater attention should be given to pulmonary development.

After birth, somatic growth and thoracic expansion occur most rapidly during the first few years of life. Prestress in the lung parenchyma is high during this period, coinciding with vigorous intra-acinar airways and alveolar formation, existing branches remodel through lengthening and dilatation. It is considered that peak growth velocity occurs between 6 months and 1 year (39).

Additionally, after birth, significant development of the distal lung takes places, as saccules begin their transformation into alveoli (40). This process, known as alveogenesis, progresses exponentially during the first 2 years of life but continues, albeit at a reduced rate, throughout adolescence (41) (Supplementary Figure 4). Alveogenesis involves the formation of secondary septa, which protrude into existing airspaces, subdividing the saccules to generate alveoli, and increasing the total surface area of the lung (42). Notably, this process requires a double-layered capillary network at the base of the newly forming septa. In parallel with alveolarization, the double-layered capillary network of the immature septa fuses to a single-layered network, optimizing the lung's gas exchange capacity (43). This process, termed microvascular maturation, follows a logarithmic and biophysical increase parallel to alveolarisation until it ceases. By the age of 2 to 3 years, approximately three-quarters of the alveolar microvasculature has matured (44) (Supplementary Figure 4).

At this point, is logical to hypothesize that before reaching full maturity, during this phase of angiogenic remodeling, the capillary network must be more fragile and thus more permeable to the entry of Mtb. This increased permeability could facilitate Mtb entry into the pulmonary veins, from where it reaches the left atrium and disseminates systemically. This mechanism may contribute to the higher risk observed in children under 2 years of age for developing miliary or meningitis TB forms. Furthermore, this dissemination strategy is reinforced as Mtb infected macrophages up-regulate the expression of genes involved in angiogenesis (45).

## 3 Does the origin of the pathogen provide any clues?

The genus *Mycobacterium* is relatively young, having emerged ~36 million years ago (46). In summary, it belongs to the phylum *Actinobacteri*a, and thus a Gram-positive bacterium that has undergone a reconstruction of its outer membrane. Not only has it been rebuilt, but it has also been enhanced through the incorporation of exceptionally large fatty acids -mycolic acids- comprising around 90 carbon atoms, in contrast to the much smaller outer membranes of Gram-negative bacteria, which contain ~12 carbon atoms. This complex structure demands substantial energy investment, resulting in a slow rate of replication.

The Mtb complex (MtbC) appears to have evolved from *M. kansasii* (47) undergoing a process of glycopeptide polarity loss within its outer membrane. Approximately 3 million years ago, this evolutionary trajectory gave rise to a *M. canetii*-like organism (48), considered the most recent ancestor of MtbC com. This gradual refinement has result in an improved ability to transmit via aerosolised droplets.

It has been hypothesized that the control of fire one million years ago played a crucial role in linking Mtb infection to early human intercessors. The use of enclosed shelters, campfires as central gathering points, and poorly ventilated spaces may have facilitated the inhalation of airborne particles, and Mtb infection (49). Indeed, evidence suggests the presence of tuberculous leptomeningitis in a fossilized frontal bound attributed to *Homo erectus*, dated to ~500,000 years ago, discovered in Kocabas, Turkey (50).

A molecular clock analysis indicates that MtbC emerged ~70,000 years ago, coinciding with the second out-of-Africa migration and Neolithic co-expansion of modern humans (51). This raises the question of the impact of Mtb infection on low-density, small-sized Paleolithic populations, which are estimated to have an effective population size of around one million individuals, organized into tribal groups of ~100 members (52).

To explore this, we developed a susceptible-exposed-infectious-recovered (SEIR) model, differentiating between smear-negative and smear-positive cases, as indicators of TB disease severity. We utilized data obtained by Ragonnet et al. from historical records of the pre-chemotherapy era (53), while also accounting for a generally better health status among Paleolithic hunter-gatherers compared to Neolithic populations. Overall, the data suggest that TB was originally a well-tolerated, highly persistent disease, even in low-density populations, posing a negligible risk for human extinction (54) (Supplementary Figure 5).

This evolutionary trajectory closely parallels that of *Helicobacter pylori*, which has coevolved with modern humans for over 50,000 years. In this case, infection was nearly universal, causing corpus-predominant gastritis, which conferred several benefits early in life, including a reduction in infectious diseases, allergy control, regulation of gastric hormones and mitigation of gastroesophageal reflux disease sequelae. Peptic ulcer disease, in contrast, is considered a modern condition (55). Should Mtb then be consider as an “old friend,” akin to non-tuberculous mycobacteria, which may have conferred protection against other infections? (56).

In this context, we can assess the impact of the Bacille Calmette-Guérin (BCG) vaccination, introduced in Sweden approximately a century ago. Its administration not only reduced TB-related mortality but also lowered mortality from other respiratory infections (57). This phenomenon has been linked to the induction of trained immunity, which has also been shown to reduce experimentally induced yellow fewer viraemia in humans (58) and to decrease respiratory infection in elderly persons (59).

More specifically, prior Mtb infection itself has been shown to protect against the development of active TB in young adult nursing students, with greater efficacy than BCG vaccination, as documented by Heimbeck at Ulleval Hospital in Oslo (60). We could also hypothesize Mtb infection to provide a heterologous protection against *Yersinia pestis* infection, originated 5,000 years ago, challenging the humankind since the Roman Empire (61).

In summary, Mtb is a non-toxic, slow-growing microorganism adapted to long-term survival in human hosts. As an evolutionarily refined parasite, its strategy is not one of overt aggression, but to develop a controlled disease in childhood causing that might favor a heterologous Th1 and trained immune response, increasing the fitness against more aggressive and acute infections, and avoiding the TB development in adults. What, then, has transformed it into a leading cause of human mortality? The key factor appears to be the dramatic shift in human lifestyles, which have become increasingly stressful and, consequently, self-destructive. Paraphrasing William Dock:

“The industrial revolution, with cheap artificial light and greatly lengthened working hours, gave TB its great harvest in Western Europe and North America. The totalitarian states have created new favorable soil by increasing the rate of urbanization and industrialization all over the world” (17).

## 4 The need for a personalized prognosis

In addressing the core challenge of TB prediction, at least three key factors must be considered in the development of an effective prognosis strategy:

### 4.1 Detecting infection vs. detecting viable bacilli

A positive tuberculin skin test (TST) or interferon-gamma release assay (IGRA) has a limited predictive value for TB development. These tests detect only memory immune responses and do not confirm the presence of viable bacilli within the body (62). In this regard a lot of efforts are being doing to detect the actual presence of Mtb using the most sensitive diagnostic tools available, thereby identifying so-called subclinical TB (28).

### 4.2 The significance of topography: reaching the upper lobes

Bacillary detection alone does not indicate whether the pathogen has colonized the upper lung lobes, where the risk of TB progression is highest. The risk is primarily associated with endogenous reinfection and the probability of internal aerosol dissemination reaching the upper lobes, as postulated by the dynamic hypothesis (63). Additionally, the risk of exogenous reinfection and the likelihood of bacillary migration to this region must also be considered (2). How feasible is it for a bacillus originating from a lesion in the lower-middle pulmonary lobes to ascend to the upper lobe? Significant research efforts are required to evaluate and quantify this risk.

Since using high-resolution images to detect lesions in the upper lobe is not realistic, more efforts should focus on determining lesion signatures in both the upper and lower lobes, as done, for instance by Marakalala et al. (64), in an attempt to identify differences in the upper lesions that could be translated to the blood level.

Most current approaches focus on the obvious spectrum of disease, tracing a unidirectional evolution of TB. However, Mtb infection is primarily a localized process. This is evidenced by the presence of lesions in different evolutionary stages within the same patient, highlighting the ongoing process of endogenous reinfection. How can this be predicted? TB progression may originate from multiple lesions, but what defines the specific lesion with the highest likelihood of evolving into active TB? Identifying this “signature lesion” is crucial for improving prognosis.

### 4.3 Major comorbid factors

Several key comorbidities are associated with an increased risk of developing active TB in adults. The most recent WHO report highlights the primary risk factors: undernutrition, alcohol abuse, smoking, diabetes, and HIV infection (65). It is evident that immunosuppressive therapies and other diseases that cause immunosuppression have also to be considered.

Another notable factor is sex-related susceptibility—why are men consistently more vulnerable to TB?(66). Year after year, across diverse geographic and socioeconomic contexts, the global male-to-female TB incidence ratio remains consistently at ~65:35 (66). Is this discrepancy driven by hormonal differences? Could it be linked to higher cortisol levels induced by stress, as has been postulated (54)? These questions require urgent clarification.

In summary, this perspective highlights the critical role of anatomical factors in TB progression and underscores the need to incorporate these variables into predictive algorithms. A comprehensive approach, integrating anatomical lesion localization, together with host comorbidities and sociodemographic risk factors, is essential for improving TB prognosis and understanding TB disease evolution.

## Data Availability

The original contributions presented in the study are included in the article/Supplementary material, further inquiries can be directed to the corresponding author.
