# Supplementary material for: Decoding the tuberculosis puzzle: mechanical factors driving disease progression
Source: Front Tuberc. 2025 Mar 28;3:1570292. doi: 10.3389/ftubr.2025.1570292 (PMC13494969; doi:10.3389/ftubr.2025.1570292)
Supplement: Supplementary file 1 [file Data_Sheet_1.pdf]

Supplementary figures

Perspective

Decoding the Tuberculosis Puzzle: Mechanical Factors Driving Disease Progression.

Pere-Joan Cardona

		HIVneg		HIVpos	
**Characteristic**	**Total No.**	**No.**	**%**	**No.**	**%**
**Cavitary lesion**	94	65	69,1	29	30,9
**Upper lobe infiltrate**	199	122	61,3	77	38,7
**Lymphadenopathy**	92	27	29,3	65	70,7
**Effusion**	66	28	42,4	38	57,6
**Effusion only**	17	4	23,5	13	76,5
**Lower or middle lung infiltrate**	142	66	46,5	76	53,5
**Miliary**	19	8	42,1	11	57,9
**Multilobar**	153	79	51,6	74	48,4
**Typical**	199	122	61,3	77	38,7

Suppl. Figure 1.- Radiographic characteristics in adult TB. Data were collected at Columbia University Medical Center between 1990 and 1999, involving patients with culture-proven pulmonary TB (n: 546). A total of 484 adult patients (83,6% < 60 years old) had radiographic data recorded in computerized charts. Data were obtained from Geng et al (5). Heatmaps range from red (highest) to green (lowest) for absolute number and from red (highest) to blue (lowest) for percentages.

Clinics	<1 y.	1-2 y.	2-5 y.	5-10 y.	>10 y.
**Pulmonary disease***	35	10	5	2	15
**Meningitis / miliary**	15	3,5	0,5	0,4	0,4
	**Gh,Lm,Br**	**Gh,Lm,Br**	**Lm,Br**	**Lm,Br,Ef,Ad**	**Ef,Ad**

* ***Pulmonary disease*** *:* **Gh**: *Ghon focus;* **Lm**: *Lymph node;* **Br**: *Bronchial;* **Ef**: *Effusion;* **Ad**: *Adult type*

Suppl. Figure 2. Natural history of childhood intrathoracic TB. The data show the percentage of clinical manifestations represented as a heatmap, with values ranging from the highest (red) to the lowest (blue). Data were obtained from Marais et al (26)

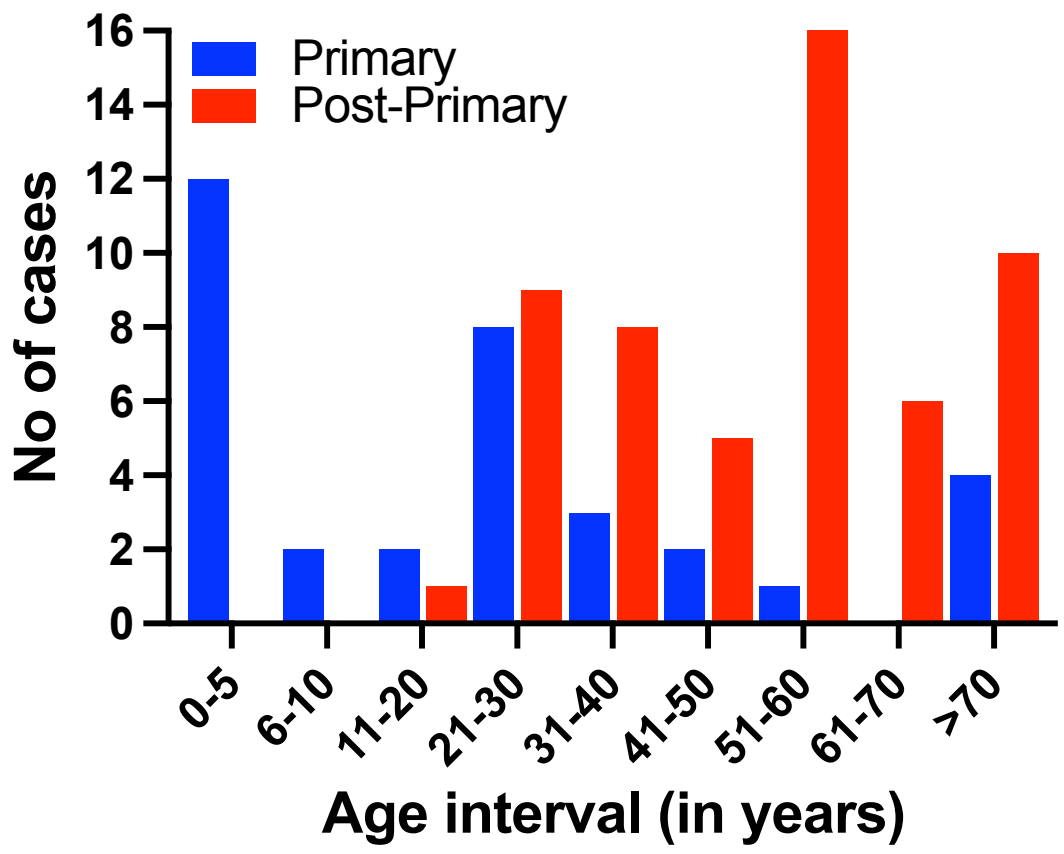

Suppl. Figure 3. Appearance of primary and post-primary TB lesions. The data show the number of patients affected by each clinical manifestation in a cohort of 91 patients. Data were obtained from Woodring et al. (29).

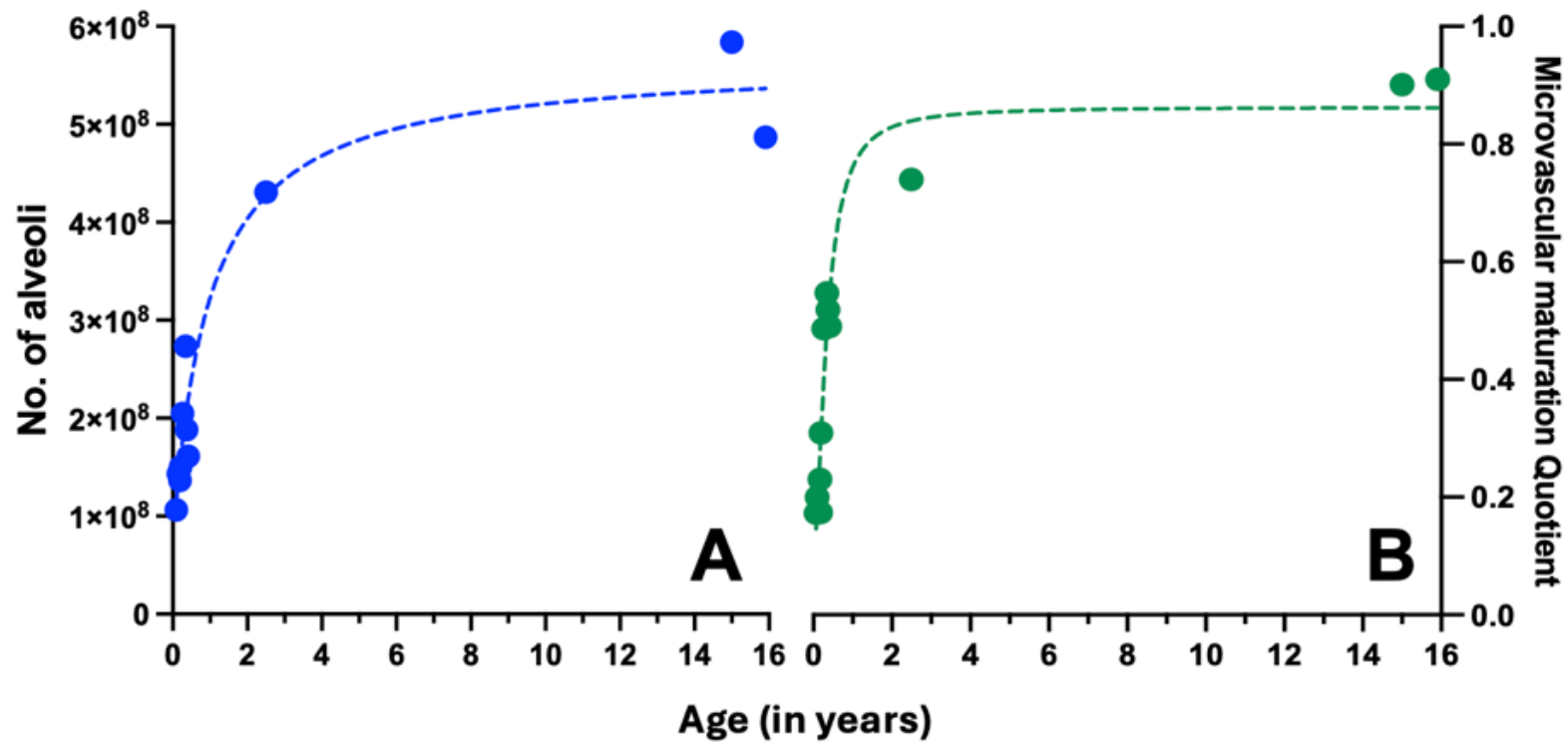

Suppl. Figure 4. Evolution of alveoli number and microvascular maturation. The data show alveolar number values obtained from Herring et al. (35) (**A**) and the microvascular maturation quotient from Schmid et al (38) (**B**). The curves represent a sigmoidal fit, while the dotted curves show a 95% confidence interval, with a R^2 of 0.9436 (**A**) and 0.9432 (**B**) (GraphPad Prism, vs 10.1.0).

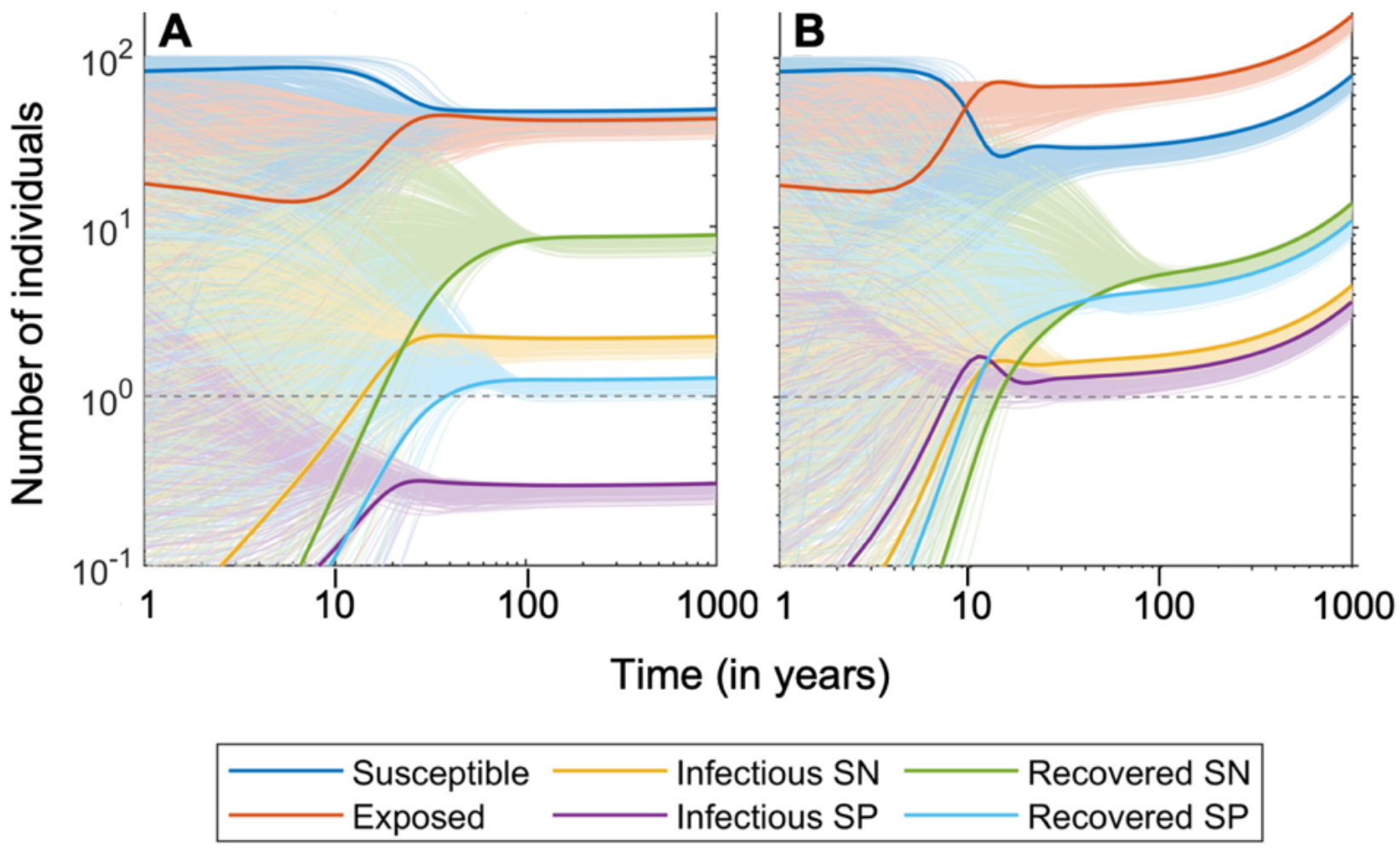

Suppl. Figure 5. Evolution of population dynamics in a SEIR model for Mtb infection (TBSpectr). The images display projections from 1000 simulations in a group remains equal to 100 individuals, with a random initial distribution across compartments. The sum of all compartments is equal to 100 throughout 1000 years of evolution, eventually reaching a stationary state. The thick lines represent the central scenario, with initial conditions set as $S_0 = 80$, $E_0 = 20$ and all other compartments starting at zero. Evolution rates are depicted, illustrating infection with modern Mtb lineages during both the Palaeolithic (A) and Neolithic (B) periods. Coloured lines represent different compartments: susceptible (blue), exposed (orange), infected smear-negative (yellow), infected smear-positive (violet), recovered smear-negative (green) and recovered smear-positive (cerulean blue). For reference, a grey dotted horizontal line marks the presence of a single individual (100 log_{10}). Modified from Cardona et al. (48).
